# Anaphylactic Rare Saponins Separated from *Panax notoginseng* Saponin and a Proteomic Approach to Their Anaphylactic Mechanism

**DOI:** 10.1155/2022/7565177

**Published:** 2022-03-11

**Authors:** Feiran Hao, Xu Pang, Kaikun Xu, Meixi Wang, Zengchun Ma, Hongling Tan, Lifeng Han, Cheng Chang, Ming Chen, Zhanwen Huang, Yue Gao

**Affiliations:** ^1^Department of Pharmaceutical Sciences, Beijing Institute of Radiation Medicine, Beijing 100850, China; ^2^State Key Laboratory of Proteomics, Beijing Proteome Research Center, Beijing Institute of Lifeomics, National Center for Protein Sciences (Beijing), Beijing 102206, China; ^3^Institute of Medicinal Biotechnology, Chinese Academy of Medical Sciences, Peking Union Medical College, Beijing 100050, China; ^4^State Key Laboratory of Component-Based Chinese Medicine, Tianjin University of Traditional Chinese Medicine, Tianjin 301617, China; ^5^Guangxi Zhongheng Innovative Pharmaceutical Research Co., Ltd., Nanning 530000, Guangxi, China

## Abstract

In recent years, many traditional Chinese medicine injections based on *Panax notoginseng* saponin (PNS) have been reported to cause anaphylaxis. Previous studies on the anaphylactic saponins of PNS and their mechanism are inadequate. In this study, potential anaphylactic saponins were obtained by the separation of PNS and preparation of each individual component through comprehensive techniques, such as liquid chromatography, preparative chromatography, HPLC, NMR, and MS. The anaphylactic abilities of these saponins were tested using RBL-2H3 cells via a *β*-hexosaminidase release rate test. The results for the mechanism of anaphylaxis were obtained by a proteomic analysis using RBL-2H3 cells. The results indicate that, among all the saponins prepared, gypenoside LXXV and notoginsenoside T5 showed strong anaphylactic abilities and notoginsenoside ST-4 and ginsenoside Rk3 showed weak anaphylactic abilities. These 4 saponins can induce anaphylaxis via direct stimulation of effector cells. The gene oncology enrichment analysis results showed that, among these saponins, only gypenoside LXXV was related to organelles of the endoplasmic reticulum and Golgi apparatus and biological processes in response to organic cyclic compounds. Four proteins in RBL-2H3 cells with the accession numbers A0A0G2JWQ0, D3ZL85, D4A5G8, and Q8K3F0 were identified as crucial proteins in the anaphylactic process. This research will help traditional Chinese medicine injection manufacturers strengthen their quality control and ensure the safety of anaphylactic saponins.

## 1. Introduction

Over the past decade, traditional Chinese medicine (TCM) injection, an innovative form of drug therapy, has become increasingly popular [1, 2]. However, according to related adverse drug reaction (ADR) reports, the ADR rate of TCM injections has risen in the past several years [3].

Related case reports about TCM injections indicate that the ADRs are primarily anaphylactoid reactions [3–5]. The clinical symptoms, including angioedema, urticaria, bronchospasm, skin flushing, hypotension, shock, and even death, typically appear over minutes to hours [6,7]. According to some previous studies, many exogenous substances including impurities and solvents can induce anaphylaxis, such as Taxol [7], Tween-80 [5, 8–10], tannic acid [5, 10], >10 kDa molecules [5, 10], Compound 48/80 (C 48/80) [11], mercuric chloride [12], drugs with ISR [12], and estrogens [12]. Some ginsenosides also have anaphylactic ability, such as ginsenoside F2 [13] and Compound K [13]. The mechanism underlying the anaphylactoid reactions is distinct from that of IgE-mediated allergies, although they both have the same symptoms. The complement system plays a key role in the process of anaphylaxis induced by many agents (e.g., radiocontrast media, liposomes, and TCM injections) [7]. Xu et al. [5, 10] studied the anaphylactoid reaction induced by Xuesaitong injection and discovered that >10 kDa molecules could activate classical complement pathways through direct stimulation to cause anaphylaxis; Tween-80 can activate the complement system through classical and alternative pathways; and tannic acid can induce anaphylactoid reactions through coactivation of the complement system, the kallikrein-kinin system, and coagulation. Another mechanism is direct stimulation, which occurs through either direct *G* protein activation or opioid receptors [7]. For example, some TCM injections can also directly induce *β*-hexosaminidase and histamine release through mast cell degranulation [7].

*Panax notoginseng* saponin (PNS), a common material for TCM injections administered to cure cardiovascular diseases, is produced from *Panax notoginseng* (Burk.) Chen [5]. ADR reports indicated that the main TCM injections based on PNS as the crude material could induce more than 12% of the total TCM injection ADRs; moreover, anaphylactoid reactions mainly occurred within 30 min after the first administration [3, 4]. However, previous studies on PNS, especially the crude PNS extract used in TCM injections, are insufficient.

To determine the specific anaphylactic constituents in PNS (from the crude extract of the root of *Panax notoginseng* (Burk.) F.H. Chen) and elucidate the mechanism, we prepared potential anaphylactic saponins via liquid chromatography, HPLC, and preparative chromatography and identified these saponins via ESI-MS and NMR. We further analyzed the cytotoxicity and *β*-hexosaminidase release rate (*β*-HexRR) of these saponins in vitro using RBL-2H3 cells. A proteomic analysis was performed to identify the possible mechanism underlying the anaphylactoid reactions.

This study will help manufacturers of TCM injections based on PNS ensure that those anaphylactic saponins are within safe levels and control the quality of TCM injections to avoid clinical anaphylactoid reactions.

## 2. Materials and Methods

### 2.1. Materials

Primary RBL-2H3 cells were purchased from American Type Culture Collection (ATCC, Manassas, VA, USA). PNS was provided by the Guangxi Wuzhou Zhongheng Pharmaceutical Corporation (Wuzhou, Guangxi, China).

Cuttable silica gel GF254 thin-layer plates were purchased from Silida Technology Co., Ltd. (Tianjin, China). MCI resin was purchased from GL Sciences (Japan). YMC chromatographic columns (Japan) were filled with reversed-phase C_18_ packings. Sequencing grade modified trypsin, dithiothreitol (DTT), and MTS cell proliferation colorimetric assay kits were purchased from Promega (Madison, WI, USA). Iodoacetamide (IAA) and trifluoroacetic acid (TFA) were purchased from Acros (Morris Plains, NJ, USA). C 48/80 and *β*-D-hexosamine were purchased from Sigma-Aldrich (St Louis, MO, USA). RIPA lysis buffer, protease inhibitor cocktail, and phosphoprotease inhibitor cocktail were purchased from CWBIO (Beijing, China). RPMI 1640 media and PBS were obtained from Gibco (Thermo Fisher, Waltham, MA, USA). Fetal bovine serum (FBS) was purchased from Biological Industries (Kibbutz Beit-Haemek, Israel).

All reagents were analytically or chromatographically pure.

### 2.2. Methods

#### 2.2.1. Separation and Preparation of the Potential Constituents of the Anaphylactoid Reaction

The following instruments were used in the experiment: ESI-MS: Thermo Fisher Q-Exactive Mass Spectrometer; NMR: Bruker DRX-500 NMR Spectrometer; HPLC: Agilent 1260 HPLC and Cosmosil 5C_18_-MS-II (4.6 × 250 mm, 5 *μ*m) analytical high-performance liquid chromatographic column; and preparative HPLC: Shimadzu LC-20AR HPLC and Agilent ZORBAX Eclipse XDB C18 (20 × 250 mm, 7 *μ*m) preparative high-performance liquid chromatographic column.

PNS (400 g) was separated using an MCI chromatographic column and eluted by 55% ethanol and 95% ethanol in series. The eluent of 95% ethanol was further separated and isocratically eluted by an ODS chromatographic column using 85% methanol. Ten out of 11 fractions were obtained, and each had a volume of 100 mL. The eleventh fraction was eluted by 100% methanol. The fractions were analyzed by HPLC and merged. Finally, 8 subfractions were obtained: Fr. 1-2, Fr. 3, Fr. 4, Fr. 5, Fr. 6, Fr. 7-8, Fr. 9-10, and Fr. 11.

#### 2.2.2. Cell Culture

RBL-2H3 cells were maintained in RPMI 1640 media with 10% FBS and 1% penicillin-streptomycin solution (100x) at 37°C and 5% CO_2_. Single-cell suspensions were plated at a density of 5-8 × 10^5^ cells/mm^2^ on a culture dish (*D* = 100 mm). When the cells reached over 80% confluence, they were harvested and passaged at a ratio of 1 : 3.

#### 2.2.3. Anaphylactic Properties of the Subfractions

Five milliliters of each subfraction was concentrated and freeze-dried in a Heidolph Rotary Evaporator (Heizbad Hei-VAP, Heidolph Instruments GmbH & CO. KG, Germany) and Biocool Freeze Dryer (Biocool FD-1C-50, Boyikang, Beijing), respectively. The obtained powders were weighed and added to the RPMI 1640 culture media with 1% DMSO, and the final concentration of each solution was 200 *μ*g/mL.

Exponentially growing RBL-2H3 cells in 6-well dishes were washed with PBS 3 times. Two milliliters of the tested supernatant was added to the corresponding wells, including the negative control group (RPMI 1640 culture media with 1% DMSO) and the positive control group (C 48/80; 20 *μ*g/mL). Each group was made in triplicate. The 6-well dishes were incubated in an incubator (37°C, 5% CO_2_) for 30 min before the supernatants were discarded. The dishes were placed under an inverted microscope (Nikon Eclipse TS100-F, Japan) for observation. The groups with high degranulation rates were considered to have the potential constituents of the anaphylactoid reaction.

#### 2.2.4. Preparation and Identification of the Potential Saponins of the Anaphylactoid Reaction

Isocratic elution and preparative separation of Fr. 3, Fr. 5, and Fr. 7-8 were conducted using preparative chromatography with mobile phases of 68% methanol/water, 74% methanol/water, and 80% methanol/water, respectively. The eluents were subsequently concentrated and freeze-dried into powders before analysis by ESI-MS and NMR for molecular weight and ^13^C-NMR information.

#### 2.2.5. IC_50_ Test

Each saponin was dissolved in RPMI 1640 media into solution or dispersed suspension. Exponentially growing RBL-2H3 cells in a 96-well dish were washed with PBS before they were incubated with the corresponding saponins. The control group only contained the culture medium and RBL-2H3 cells, while the blank group only contained the culture medium. Cells were incubated for 24 h, and the supernatants were discarded. After washing with PBS 3 times, 100 *μ*L culture medium was added to each well, and then MTS reagents were added for an incubation of approximately 30 min. The dish was subsequently placed on a microplate reader to record the absorbance.

#### 2.2.6. *β*-Hexosaminidase Release Rate

Exponentially growing RBL-2H3 cells in 24-well dishes were washed with RPMI 1640 media 3 times. Then, 100 *μ*L of RPMI 1640 medium was added to each well before incubation for 10 min. Another 100 *μ*L of each tested constituent at different concentrations was added to the corresponding wells. Incubations of 15 min, 30 min, 1 h, and 2 h were conducted before an ice bath for 10 min to end the reaction. Fifty microliters of the supernatant in each well together with the substrate (*β*-D-hexosamine; 4 mM) were added to a 96-well dish before incubation for 1 h at 37°C. Then, 150 *μ*L of stop solution (glycine buffer; pH 10.7; 200 mM) was added to each well to stop the reaction. The absorbance (absorbance of supernatant, AoS) at 405 nm was measured for all wells. The rest of the supernatant in the 24-well dishes was removed, and 200 *μ*L of 0.5% Triton X-100 was added to each well to lyse the cells. The lysate of each well was centrifuged at 3000 rpm for 1 min, and 50 *μ*L of the supernatant in each well was transferred to a 96-well dish. *β*-D-Hexosamine (4 mM) as the substrate was added to each well at the same time. The 96-well dish was incubated at 37°C for 1 h before 150 *μ*L of stop solution was added to each well. The absorbance (absorbance of lysate, AoL) at 405 nm was measured for all wells. *β*-HexRR can be calculated using the following equation:

(1) $$\begin{array}{c} \beta -HexRR=\frac{AoS-blank_{AoS}}{\left(AoL-blank_{AoL}\right)+\left(AoS-blank_{AoS}\right)}\times 100\%. \end{array}$$

#### 2.2.7. Proteomic Analysis

RBL-2H3 cells of different groups were collected into the corresponding microtubes and centrifuged at 4000 rpm for 4 min before the supernatant was discarded. The cells were washed with PBS and centrifuged once again, and the supernatant was discarded. RIPA lysis buffer and protease inhibitor were added before treatment in an ice bath for 30 min. The cells were then lysed via ultrasonication. Protein concentrations were measured using the bicinchoninic acid (BCA) method.

Urea (8 M) and DTT (final concentration 10 mM) were added to the microtubes containing the cell lysate at volume ratios of 1 : 1 and 1 : 2, respectively. The mixture was incubated at 37°C for 4 h before IAA was added (IAA : DTT = 5 : 1, mol/mol). The mixture was further incubated in the dark for 1 h at room temperature. NH_4_HCO_3_ (50 mM) was added to the mixture to dilute urea to below 1 M. Sequencing grade modified trypsin was added (trypsin: protein = 1 : 50, w/w). The mixture was incubated in a water bath at 37°C for 10–16 h.

The mixture was desalted before the peptide concentration was measured via a NanoDrop (Thermo Fisher, Waltham, MA, USA). LC-MS/MS analysis was performed on a Q-Exactive HF Hybrid Quadrupole-Orbitrap mass spectrometer (Thermo Fisher Scientific) coupled online to a nanoflow LC system (EASY-nLC 1000, Thermo Fisher Scientific). Peptides were delivered onto a 2 cm self-packed trap column (100 *μ*m inner diameter, 3 *μ*m resin, ReproSil-Pur C18-AQ, Dr Maisch GmbH) in solvent A (0.1% formic acid FA in HPLC grade water). After loading and washing, the peptides were transferred to a 12 cm column (150 *μ*m inner diameter, 1.9 *μ*m resin, ReproSil-Pur C18-AQ, Dr Maisch GmbH) and separated over 78 min nonlinear gradients from 6% to 95% solvent B (0.1% FA in ACN) at a flow rate of 600 nL/min. The exact elution gradients were as follows: 0–16 min, 6%–10% B; 16–51 min, 10%–24% B; 51–71 min, 24%–34% B; 71–72 min, 34%–95% B; 72–78 min, 95% B.

The peptides were ionized using a 2.0 kV spray voltage and a capillary temperature of 320°C. The data acquisition was performed in the OT–IT mode. Full MS scans (300 to 1,400 m/z) were performed at a resolution of 120,000, a maximum injection time of 80 ms, and an AGC target value of 3*e*6. Tandem mass spectra were generated for up to 20 precursors by HCD with a normalized collision energy of 27%. The dynamic exclusion was set to 12 s. The MS2 spectra were read out in the Orbitrap at a resolution of 15,000 with an AGC target value of 5*e*4 and a maximum injection time of 19 ms.

#### 2.2.8. Data Analysis

The raw files were searched by Thermo Proteome Discoverer (PD) (2.1.1.21) software against the protein database uniprot_rat_170221, which contains 8094 proteins. The parameters and settings were as follows: precursor mass tolerance, 15 ppm; fragment mass tolerance, 20 mmu; enzyme name, trypsin; max missed cleavage sites, 2; static modification, carbamidomethyl; dynamic modifications, acetyl and oxidation. The confidence level was set to 95%.

Differentially expressed proteins were determined by the ratio of protein expression of the adjacent groups. The expression of upregulated proteins in the high dose group/medium dose group, medium dose group/low dose group, and low dose group/control group was all above 1.2; downregulated proteins in the high dose group/medium dose group, medium dose group/low dose group, and low dose group/control group were all below 1/1.2. Cluster heatmaps were drawn based on the upregulated proteins and downregulated proteins. Gene Oncology (GO) and Kyoto Encyclopedia of Genes and Genomes (KEGG) enrichment analyses were based on the upregulated proteins, downregulated proteins, and the DAVID (Database for Annotation, Visualization and Integrated Discovery) tool.

## 3. Results and Discussion

### 3.1. Anaphylactic Properties of the Subfractions

**Figure 1:**
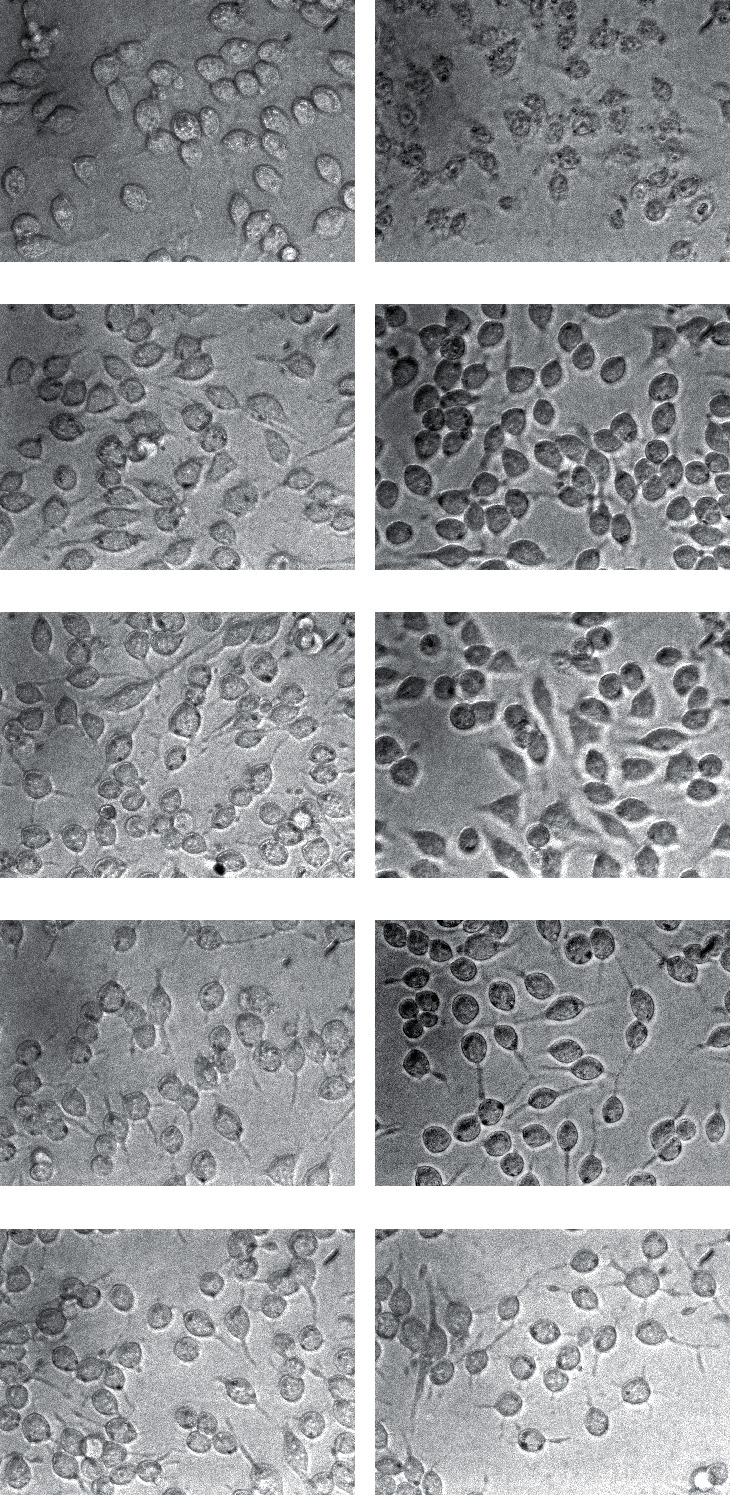
Anaphylactic property test results of each subfraction. The anaphylactic property was tested by the observation of RBL-2H3 cells incubated with each subfraction using an inverted microscope (200x). RBL-2H3 cells showed no degranulation in (a) the negative control group and strong degranulation (blurred cell membrane and obvious granules) in (b) the positive control group. Cells in the group of (d) Fr. 3, (f) Fr. 5, and (h) Fr. 7-8 showed anaphylactoid reactions (>50% degranulated cells). Cells in the group of (c) Fr. 1-2, (e) Fr. 4, (g) Fr. 6, (i) Fr. 9-10, and (j) Fr. 11 showed no anaphylactoid reactions (<30% degranulated cells).

Photographs of RBL-2H3 cells incubated with each subfraction are presented in Figure 1. The cells in the positive control groups were strongly degranulated and showed blurred cell membranes and obvious granules. In contrast, few cells in the negative control groups showed degranulation. Fr. 3, Fr. 5, and Fr. 7-8 were shown to induce degranulation of RBL-2H3 cells (>50% degranulated cells) while Fr. 1-2, Fr. 4, Fr. 6, Fr. 9-10, and Fr. 11 showed no anaphylactic properties.

### 3.2. Structural Identification of the Potential Saponins of the Anaphylactoid Reaction

Compounds 1, 2, and 3 were obtained from the eluents of Fr. 3. Compounds 2, 3, and 4 were obtained from the eluents of Fr. 5. Compounds 5, 6, and 7 were obtained from the eluents of Fr. 7-8. All compounds were white amorphous powders that showed a red color on thin-layer plates when reacting with 10% ethanol sulfate solution. The ^13^C-NMR spectra are shown in Figure S1, and the data are listed in Supplementary Materials. The data for compounds 1 and 4 were in accordance with the data in [14], and they were identified as notoginsenoside T5 (noto-T5) and notoginsenoside T4 (noto-T3), respectively. The data for compounds 2 and 3 were in accordance with the data in [15]; therefore, they were identified as ginsenoside Rk3 (Rk3) and ginsenoside Rh4 (Rh4), respectively. The data for compounds 5, 6, and 7 were in accordance with the data in [16–18], respectively; therefore, they were identified as ginsenoside Rg3 (Rg3), gypenoside LXXV (gyp-LXXV), and notoginsenoside ST-4 (noto-ST-4), respectively. Notably, all of the above saponins are rare saponins in PNS.

### 3.3. Basic Physical and Chemical Information of Each Saponin

Saponins on a microgram scale were weighed and mixed with 1 mL of RPMI 1640 media with 1% DMSO at room temperature. The chemical structure, chemical formula, molecular weight, and solubility of each saponin are shown in Table 1.

Noto-T3 was not involved in the following studies because it presented flocculent precipitate and aggregation in the solution and was hard to be solved or shattered into small pieces, which was not suitable for the follow-up experiments.

### 3.4. IC_50_ Test

To prepare an appropriate concentration gradient of each saponin, an IC_50_ test was performed based on RBL-2H3 cells using the MTS assay. The average cell viability of each group is shown in Table S1.

The IC_50_ value of each saponin was obtained by linear interpolation. According to the IC_50_ value of each saponin, the concentrations of the low, medium, and high dose groups were set geometrically, following the rules that the dose of high dose groups was close to but lower than the IC_50_ value (Table 2). According to the experiments, C 48/80 could induce abnormal absorbance in the IC_50_ test. Therefore, the low, medium, and high dose groups of C 48/80 were set based on [12].

### 3.5. *β*-Hexosaminidase Release Rate

**Figure 2:**
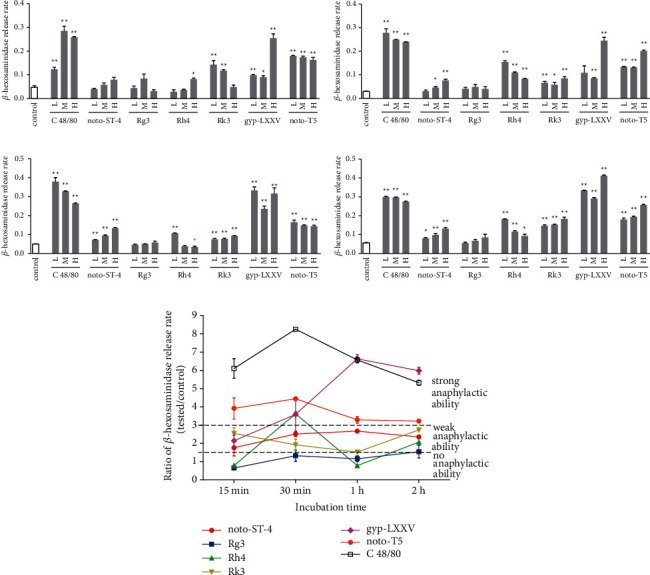
*β*-HexRRs of all tested groups and the anaphylactic ability of each saponin. *β*-HexRRs of the control groups, C 48/80 groups, noto-ST-4 groups, Rg3 groups, Rh4 groups, Rk3 groups, gyp-LXXV groups, and noto-T5 groups were tested with different incubation times of (a) 15 min; (b) 30 min; (c) 1 h; and (d) 2 h. (e) Anaphylactic ability of each saponin at a concentration of 0.03 *μ*mol/mL was determined by the *β*-HexRRs of the control groups and C 48/80 groups. L = low dose group, M = medium dose group, and H = high dose group. The concentrations of each low dose group, medium dose group, and high dose group are shown in Table 2. Each group was repeated 3 times, and the data are expressed as the mean ± SEM (*n* = 3). ^*∗*^*p* < 0.05 and ^*∗∗*^*p* < 0.01, compared with the corresponding control group (*t*-test). The *β*-HexRR of the high dose group of Rh4 with an incubation time of 1 h was significantly lower than that of the control group, while all the other significant differences were significantly higher.

The *β*-hexosaminidase release rate is the gold standard to quantitatively evaluate the level of anaphylactoid reactions [7, 10, 19]. According to clinical reports, anaphylactoid reactions induced by TCM injections containing PNS mainly occur within 30 min after administration, and few ADR cases still occur within 30 min to 2 h after administration [20]. Therefore, 4 periods of time (15 min, 30 min, 1 h, and 2 h) were set to investigate the *β*-HexRR of the control group, C 48/80 group, and 6 saponin groups (Figures 2(a)–2(d)).

The results indicated that, compared with the control and C 48/80 groups, Rg3 had no anaphylactic ability, gyp-LXXV and noto-T5 had strong anaphylactic abilities, and noto-ST-4 and Rk3 had weak anaphylactic abilities (Figure 2(e)). For Rh4, the results cannot support the conclusion that it possesses anaphylactic ability. Surprisingly, its high dose group with an incubation time of 1 h even induced a significantly lower *β*-HexRR.

These results also demonstrated that gyp-LXXV, noto-T5, noto-ST-4, and Rk3 can directly activate *β*-hexosaminidase release and degranulation from RBL-2H3 cells, which suggested that these saponins can induce anaphylaxis via direct stimulation of effector cells.

### 3.6. Proteomic Analysis

**Figure 3:**
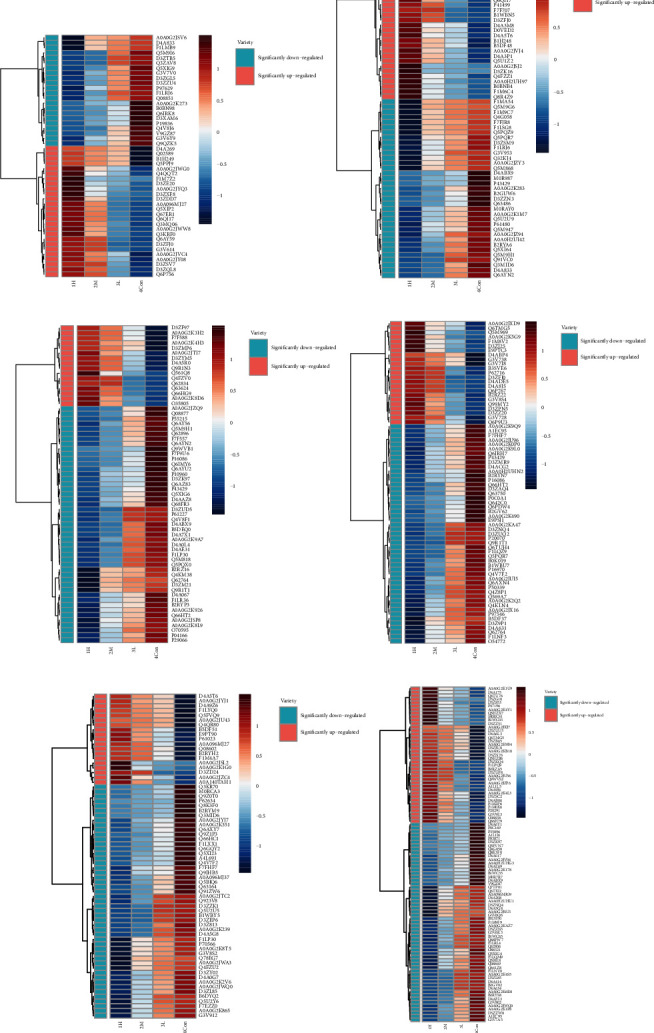
Cluster heatmaps of the significantly upregulated and downregulated proteins of the (a) noto-ST-4 groups, (b) Rg3 groups, (c) Rh4 groups, (d) Rk3 groups, (e) gyp-LXXV groups, and (f) noto-T5 groups with an incubation time of 30 min.

**Figure 4:**
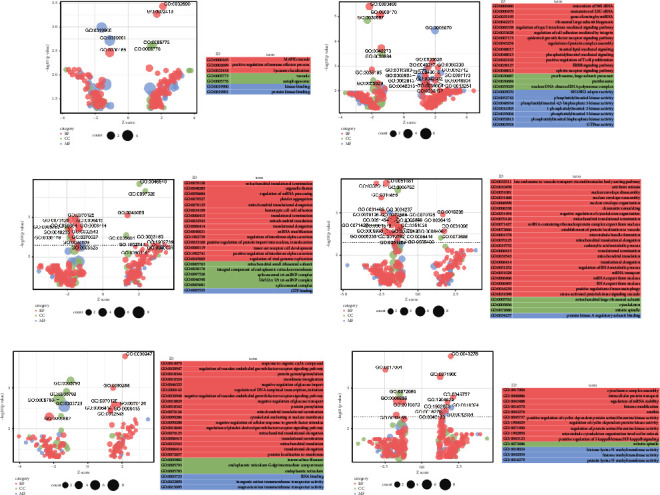
GO analysis results of BP, CC, and MF of the (a) noto-ST-4 groups, (b) Rg3 groups, (c) Rh4 groups, (d) Rk3 groups, (e) gyp-LXXV groups, and (f) noto-T5 groups with an incubation time of 30 min.

To investigate the mechanism of the anaphylactoid reaction induced by the potential anaphylactic saponins in PNS, differentially expressed proteins of each saponin group with an incubation time of 30 min were studied. According to the filter conditions, proteins were screened and classified into upregulated proteins and downregulated proteins (Table 3). Based on the upregulated and downregulated proteins, cluster heatmaps were drawn (Figure 3); GO and KEGG enrichment analyses were also carried out via the DAVID tool. GO enrichment analyses of the biological process (BP) (*p* < 0.005), cellular component (CC) (*p* < 0.005), and molecular function (MF) (*p* < 0.005) categories are displayed in Figure 4. The KEGG enrichment pathway analysis results are shown in Figure S2.

## 4. Discussion

The *β*-HexRR results were in accordance with a previous study showing that Rg3 could inhibit mast cell-mediated allergies by blocking degranulation [21].

**Figure 5:**
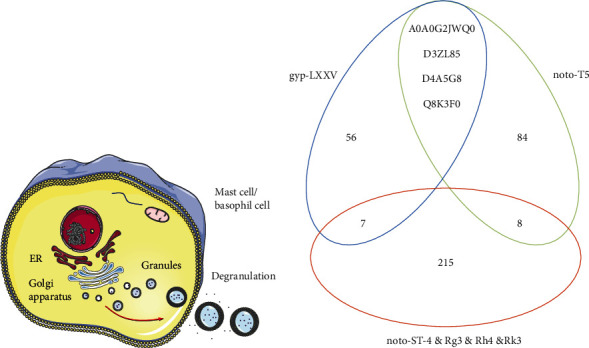
Illustration of the anaphylactoid reactions in RBL-2H3 cells induced by gyp-LXXV and noto-T5; (b) Venn diagram of the overlapping proteins of the gyp-LXXV groups, noto-T5 groups, and the other 4 groups.

Theoretical studies have suggested that anaphylactoid reactions are most closely related to the endoplasmic reticulum and Golgi apparatus, which are the most crucial organelles in the formation of granules (Figure 5(a)) [19]. Our results of the gyp-LXXV groups were consistent with this conclusion. According to the GO enrichment analysis results of CC, only the gyp-LXXV groups, which present strong anaphylactic abilities, are related to the endoplasmic reticulum (GO:0005793, GO:0005783) and Golgi apparatus (GO:0005793). Other groups are only related to certain common organelles such as the nucleus, mitochondrion, and cytosol, which may participate in almost every biological process of cell activities.

According to the GO enrichment analysis results of BP, only the gyp-LXXV groups are related to the response to organic cyclic compound (GO:0014070), which indicates that gyp-LXXV (organic cyclic compounds) could trigger these responses and finally induce anaphylaxis.

Further analysis demonstrated that there were 4 proteins in common in the differentially expressed proteins of the strong anaphylactic groups (gyp-LXXV groups and noto-T5 groups), namely, A0A0G2JWQ0, D3ZL85, D4A5G8, and Q8K3F0, all of which were downregulated proteins and not included in the differentially expressed proteins of the other 4 groups (Figure 5(b)). This result indicated that these 4 proteins had a higher possibility of playing a key role in the anaphylactoid reactions of RBL-2H3 cells.

For gyp-LXXV, KEGG enrichment pathway analysis results were not obtained after data filtration. For the noto-T5 and other 4 groups, the KEGG enrichment pathway analysis result was not consistent with RBL-2H3 cells and anaphylactoid reactions (Figure S1). The results indicate that the molecular mechanism of the anaphylactoid reactions induced by gyp-LXXV and noto-T5 requires further in-depth study.

Although gyp-LXXV and noto-T5 were identified as strong anaphylactic saponins and noto-ST-4 and Rk3 were identified as weak anaphylactic saponins, these findings do not necessarily mean that every TCM injection based on PNS will induce anaphylaxis. In fact, incomplete clinical reports indicated that anaphylactoid reactions induced by several main TCM injections based on PNS had an overall incidence rate of less than 1% and varied according to the batch [20, 22], which also indicates that anaphylactic saponins are most likely to be rare saponins and that the occurrence of anaphylaxis depends on the total content of these rare saponins. Other related factors include the dripping speed, age, sex, and body constitution [3, 4]. Here we only discuss the potential anaphylactic abilities of these saponins and offer a guide for TCM injection manufacturers.

## 5. Conclusions

In conclusion, among the 7 saponins identified, gyp-LXXV and noto-T5 had strong anaphylactic abilities, noto-ST-4 and Rk3 had weak anaphylactic abilities, and Rg3 had no anaphylactic ability. Anaphylactic saponins can induce anaphylaxis via direct stimulation of effector cells. The results of proteomic studies indicated that the anaphylactoid reaction of gyp-LXXV was related to the biological processes in the response to organic cyclic compounds and the cellular components of the endoplasmic reticulum and Golgi apparatus. Four proteins were most likely to play key roles in the anaphylactoid reactions of RBL-2H3 cells, and their accession numbers were A0A0G2JWQ0, D3ZL85, D4A5G8, and Q8K3F0. To avoid clinical anaphylactoid reactions, manufacturers should strictly control the ingredients of TCM injections based on PNS and ensure that gyp-LXXV, noto-T5, noto-ST-4, and Rk3 are within safe levels.

## Figures and Tables

**Table 1:** Name, chemical structure, chemical formula, molecular weight, and solubility of each potential anaphylactic saponin.

Name	Structure	Chemical formula and molecular weight	Solubility (in RPMI 1640 media with 1% DMSO)
Noto-T5	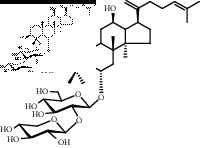	C_41_H_68_O_12_	Soluble
		752	
Rk3	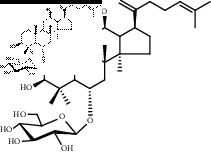	C_36_H_60_O_8_	Soluble
		620	
Rh4	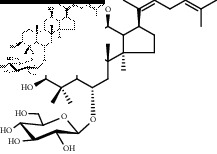	C_36_H_60_O_8_	Soluble
		620	
Noto-T3	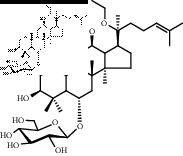	C_38_H_66_O_9_	Insoluble, flocculent precipitate, and aggregation
		666	
Rg3	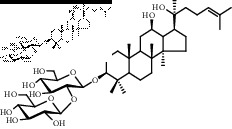	C_42_H_72_O_13_	Soluble
		784	
Gyp-LXXV	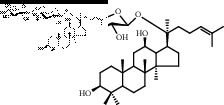	C_42_H_72_O_13_	Soluble
		784	
Noto-ST-4	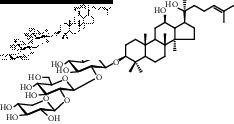	C_47_H_80_O_17_	Soluble
		916	

**Table 2:** IC_50_ values and concentrations of the low, medium, and high dose groups of each potential anaphylactic saponin.

	IC_50_	Low	Medium	High
Noto-ST-4 (mM)	0.040	0.008	0.015	0.030
Rg3 (mM)	0.040	0.008	0.015	0.030
Rh4 (mM)	0.075	0.015	0.030	0.060
Rk3 (mM)	0.085	0.015	0.030	0.060
Gyp-LXXV (mM)	0.140	0.030	0.060	0.120
Noto-T5 (mM)	0.140	0.030	0.060	0.120
C 48/80 (*μ*g/mL)	—	10	20	40

**Table 3:** Number of upregulated and downregulated proteins and total identified proteins of each saponin group with an incubation time of 30 min.

	Upregulated	Downregulated	Total
Noto-ST-4	26	22	4372
Rg3	27	35	4413
Rh4	16	47	4253
Rk3	23	49	4337
Gyp-LXXV	19	49	5016
Noto-T5	39	57	5025

## Data Availability

All data used to support the findings of this study are available from the corresponding author upon request.

## Supplementary Materials

Supplementary Materials The Supplementary Materials contain the ^13^C-NMR spectrum data of compounds 1–7 (Figure S1), IC_50_ test results (Table S1), the detailed information about the upregulated and downregulated proteins (in 6 pdf files), and KEGG enrichment pathway analysis results (Figure S2).

## References

[1] Jiang C., Shen J., Shou D. (2019). Identification of high-risk patients for ADR induced by traditional Chinese medicine injection: a nested case-control study. *Scientific Reports*.

[2] Yang Z.-R., Wang Z.-H., Tang J.-F. (2018). UPLC-QTOF/MSE and bioassay are available approaches for identifying quality fluctuation of Xueshuantong lyophilized powder in clinic. *Frontiers in Pharmacology*.

[3] Li H., Wang S., Yue Z., Ren X., Xia J. (2018). Traditional Chinese herbal injection: current status and future perspectives. *Fitoterapia*.

[4] Li H., Deng J., Deng L., Ren X., Xia J. (2019). Safety profile of traditional Chinese herbal injection: an analysis of a spontaneous reporting system in China. *Pharmacoepidemiology and Drug Safety*.

[5] Xu Y., Dou D., Ran X., Liu C., Chen J. (2015). Integrative analysis of proteomics and metabolomics of anaphylactoid reaction induced by Xuesaitong injection. *Journal of Chromatography A*.

[6] Simons F. E. R., Ardusso L. R. F., Bilò M. B. (2012). 2012 Update: World Allergy Organization Guidelines for the assessment and management of anaphylaxis. *Current Opinion in Allergy & Clinical Immunology*.

[7] Zhang B., Li Q., Shi C., Zhang X. (2018). Drug-induced pseudoallergy: a review of the causes and mechanisms. *Pharmacology*.

[8] Mi Y.-N., Ping N.-N., Xiao X., Zhu Y.-B., Liu J., Cao Y.-X. (2014). The severe adverse reaction to vitamin K1 injection is anaphylactoid reaction but not anaphylaxis. *PLoS One*.

[9] Yang R., Lao Q.-C., Yu H.-P. (2015). Tween-80 and impurity induce anaphylactoid reaction in zebrafish. *Journal of Applied Toxicology*.

[10] Xu Y., Liu C., Dou D., Wang Q. (2017). Evaluation of anaphylactoid constituents in vitro and in vivo. *International Immunopharmacology*.

[11] Palomäki V. A. B., Laitinen J. T. (2016). The basic secretagogue compound 48/80 activates G proteins indirectly via stimulation of phospholipase D–lysophosphatidic acid receptor axis and 5-HT_1A_ receptors in rat brain sections. *British Journal of Pharmacology*.

[12] Yu Y., Blokhuis B. R., Garssen J., Redegeld F. A. (2016). Non-IgE mediated mast cell activation. *European Journal of Pharmacology*.

[13] Wang L., Zhang F., Cao Z. (2017). Ginsenoside F2 induces the release of mediators associated with Anaphylactoid reactions. *Fitoterapia*.

[14] Teng R.-W., Li H.-Z., Wang D.-Z., Yang C.-R. (2004). Hydrolytic reaction of plant extracts to generate molecular diversity: new dammarane glycosides from the mild acid hydrolysate of root saponins of panax notoginseng. *Helvetica Chimica Acta*.

[15] Park I. H., Kim N. Y., Han S. B. (2002). Three new dammarane glycosides from heat processed ginseng. *Archives of Pharmacal Research*.

[16] Yang X., Li K., Zhou Q. (2015). 20(S)-Ginsenoside-Rf2, a novel triterpenoid saponin from stems and leaves of Panax ginseng. *Chinese Traditional and Herbal Drugs*.

[17] Kazuko Y., Masahiro A., Kuki K., Tsunematsu T., Shigenobu A. (1987). Studies on the constituents of cucurbitaceae plants. XVIII. On the saponin constituents of gynostemma pentaphyllum MAKINO.(13). *Yakugaku Zasshi*.

[18] Pei Y., Du Q., Liao P. (2011). Notoginsenoside ST-4 inhibits virus penetration of herpes simplex virus in vitro. *Journal of Asian Natural Products Research*.

[19] Wernersson S., Pejler G. (2014). Mast cell secretory granules: armed for battle. *Nature Reviews Immunology*.

[20] Peng H., Li G., Zhang W. (2017). Cases analysis and mechanism of 19 cases of adverse drug reactions induced by Xueshuantong injection. *China Pharmaceuticals*.

[21] Kee J.-Y., Hong S.-H. (2019). Ginsenoside Rg3 suppresses mast cell-mediated allergic inflammation via mitogen-activated protein kinase signaling pathway. *Journal of Ginseng Research*.

[22] Jin X., Wang K., Zhai J. (2020). Analysis of Injection of Xuesaitong (lyophilized) in 30097 cases in real world. *China Journal of Chinese Materia Medica*.

