# Supplementary material for: Anaphylactic Rare Saponins Separated from Panax notoginseng Saponin and a Proteomic Approach to Their Anaphylactic Mechanism
Source: Evid Based Complement Alternat Med. 2022 Mar 11;2022:7565177. doi: 10.1155/2022/7565177 (PMC8933111; doi:10.1155/2022/7565177)
Supplement: Supplementary Materials — The Supplementary Materials contain the 13C-NMR spectrum data of compounds 1–7 (Figure S1), IC50 test results (Table S1), the detailed information about the upregulated and downregulated proteins (in 6 pdf files), and KEGG enrichment pathway analysis results (Figure S2). [file 7565177.f1.docx]

**Supplementary Materials**

The ^13^C-NMR spectrum data of compound 1-7:

Compound 1: ^13^C-NMR (pydrine-d5, 125 MHz) δ: 39.6(C-1), 27.8(C-2), 78.8(C-3), 40.3(C-4), 61.4(C-5), 80.3(C-6), 45.2(C-7), 41.3(C-8), 52.1(C-9), 39.7(C-10), 32.8(C-11), 72.5(C-12), 50.6(C-13), 51.2(C-14), 32.6(C-15), 27.1(C-16), 48.3(C-17), 17.4(C-18), 17.8(C-19), 155.5(C-20), 108.1(C-21), 33.8(C-22), 30.7(C-23), 125.4(C-24), 131.2(C-25), 25.8(C-26), 17.8(C-27), 31.7(C-28), 16.7(C-29), 16.8(C-30), 103.6(C-1′), 79.5(C-2′), 79.9(C-3′), 71.8(C-4′), 78.1(C-5′), 63.0(C-6′), 104.9(C-1′′), 75.9(C-2′′), 78.8(C-3′′), 71.3(C-4′′), 67.3(C-5′′).

Compound 2: ^13^C-NMR (pydrine-d5, 125 MHz) δ: 39.6(C-1), 28.0(C-2), 78.6(C-3), 41.3(C-4), 61.5(C-5), 80.1(C-6), 45.4(C-7), 41.3(C-8), 50.7(C-9), 39.8(C-10), 32.8(C-11), 72.5(C-12), 52.1(C-13), 51.2(C-14), 32.5(C-15), 30.7(C-16), 48.3(C-17), 17.4(C-18), 17.8(C-19), 155.5(C-20), 108.2(C-21), 33.8(C-22), 27.1(C-23), 125.4(C-24), 131.2(C-25), 25.8(C-26), 17.8(C-27), 31.7(C-28), 16.4(C-29), 16.8(C-30), 106.0(C-1′), 75.5(C-2′), 79.7(C-3′), 71.9(C-4′), 78.1(C-5′), 63.1(C-6′).

Compound 3: ^13^C-NMR (pydrine-d5, 125 MHz) δ: 39.5(C-1), 28.0(C-2), 78.6(C-3), 41.4(C-4), 61.5(C-5), 80.1(C-6), 45.4(C-7), 41.4(C-8), 50.7(C-9), 39.8(C-10), 32.6(C-11), 72.6(C-12), 50.4(C-13), 50.9(C-14), 32.3(C-15), 28.8(C-16), 50.6(C-17), 17.4(C-18), 17.8(C-19), 140.1(C-20), 13.1(C-21), 123.5(C-22), 27.5(C-23), 123.9(C-24), 131.3(C-25), 25.7(C-26), 17.7(C-27), 31.8(C-28), 16.4(C-29), 16.8(C-30), 106.0(C-1′), 75.5(C-2′), 79.7(C-3′), 71.9(C-4′), 78.2(C-5′), 63.1(C-6′).

Compound 4: ^13^C-NMR (pydrine-d5, 125 MHz) δ: 39.7(C-1), 28.0(C-2), 78.6(C-3), 40.4(C-4), 61.5(C-5), 80.1(C-6), 45.2(C-7), 41.2(C-8), 50.0(C-9), 39.5(C-10), 31.4(C-11), 70.5(C-12), 49.4(C-13), 51.7(C-14), 31.2(C-15), 26.2(C-16), 47.4(C-17), 17.4(C-18), 17.7(C-19), 80.0(C-20), 19.1(C-21), 36.4(C-22), 21.8(C-23), 125.0(C-24), 131.2(C-25), 25.8(C-26), 17.7(C-27), 31.8(C-28), 16.4(C-29), 17.2(C-30), 56.5(CH_3_CH_2_O-), 15.6(CH_3_CH_2_O-), 106.0(C-1′), 75.5(C-2′), 79.7(C-3′), 71.9(C-4′), 78.2(C-5′), 63.2(C-6′).

Compound 5: ^13^C-NMR (pydrine-d5, 125 MHz) δ: 39.7(C-1), 26.8(C-2), 88.9(C-3), 39.2(C-4), 56.4(C-5), 18.5(C-6), 35.2(C-7), 40.0(C-8), 50.4(C-9), 37.0(C-10), 32.1(C-11), 71.0(C-12), 48.6(C-13), 51.7(C-14), 31.4(C-15), 26.9(C-16), 54.8(C-17), 15.9(C-18), 16.4(C-19), 73.0(C-20), 27.1(C-21), 35.9(C-22), 23.0(C-23), 126.3(C-24), 130.7(C-25), 25.8(C-26), 17.7(C-27), 28.2(C-28), 16.6(C-29), 17.0(C-30), 105.1(C-1′), 83.5(C-2′), 78.3(C-3′), 71.7(C-4′), 78.1(C-5′), 62.9(C-6′), 106.1(C-1′′), 77.2(C-2′′), 78.0(C-3′′), 71.7(C-4′′), 78.4(C-5′′), 62.8(C-6′′).

Compound 6: ^13^C-NMR (pydrine-d5, 125 MHz) δ: 39.6(C-1), 28.7(C-2), 78.1(C-3), 39.6(C-4), 56.4(C-5), 18.8(C-6), 35.2(C-7), 40.1(C-8), 50.4(C-9), 37.5(C-10), 30.9(C-11), 70.2(C-12), 49.6(C-13), 51.4(C-14), 30.8(C-15), 26.7(C-16), 51.7(C-17), 16.1(C-18), 16.3(C-19), 83.5(C-20), 22.4(C-21), 36.3(C-22), 23.2(C-23), 126.0(C-24), 131.1(C-25), 25.8(C-26), 18.0(C-27), 28.3(C-28), 16.4(C-29), 17.5(C-30), 98.1(C-1′), 74.9(C-2′), 79.3(C-3′), 71.8(C-4′), 77.1(C-5′), 70.3(C-6′), 105.4(C-1′′), 75.3(C-2′′), 78.4(C-3′′), 71.7(C-4′′), 78.4(C-5′′), 62.9(C-6′′).

Compound 7: ^13^C-NMR (pydrine-d5, 125 MHz) δ: 39.8(C-1), 26.8(C-2), 88.9(C-3), 39.2(C-4), 56.4(C-5), 18.5(C-6), 35.2(C-7), 40.0(C-8), 50.4(C-9), 37.0(C-10), 32.1(C-11), 71.0(C-12), 48.6(C-13), 51.7(C-14), 31.4(C-15), 26.9(C-16), 54.8(C-17), 15.9(C-18), 16.4(C-19), 73.0(C-20), 27.1(C-21), 35.9(C-22), 23.0(C-23), 126.4(C-24), 130.7(C-25), 25.8(C-26), 17.7(C-27), 28.2(C-28), 16.6(C-29), 17.0(C-30), 104.8(C-1′), 83.1(C-2′), 77.8(C-3′), 71.9(C-4′), 77.7(C-5′), 63.0(C-6′), 103.2(C-1′′), 84.6(C-2′′), 78.7(C-3′′), 71.1(C-4′′), 78.0(C-5′′), 62.9(C-6′′), 106.5(C-1′′′), 76.0(C-2′′′), 78.3(C-3′′′), 70.7(C-4′′′), 67.5(C-5′′′).


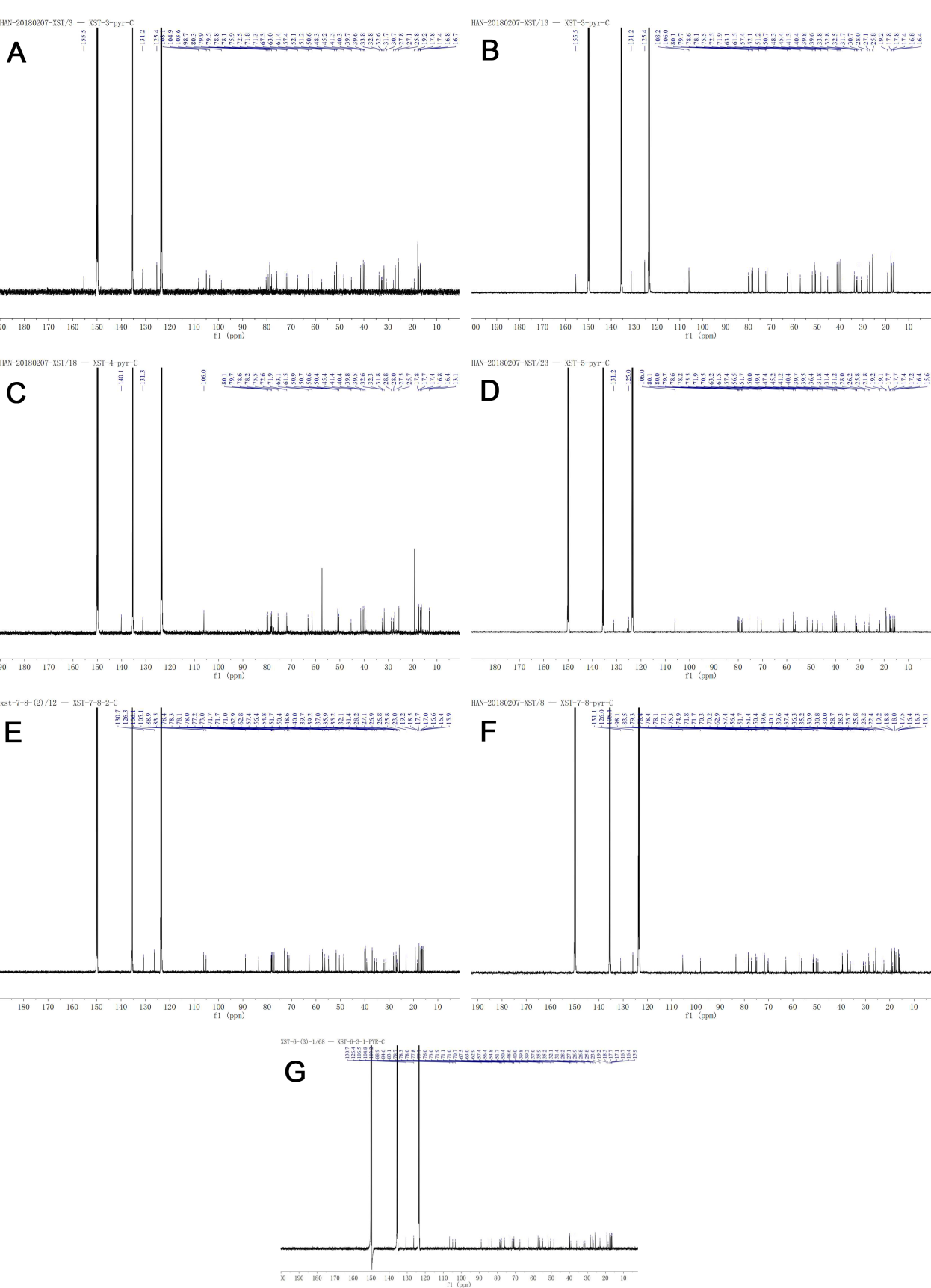


**Figure S1** ^13^C-NMR spectra of (A) compound 1 (noto-T5), (B) compound 2 (Rk3), (C) compound 3 (Rh4), (D) compound 4 (noto-T3), (E) compound 5 (Rg3), (F) compound 6 (gyp-LXXV) and (G) compound 7 (noto-ST-4).

**Table S1** Average cell viability of each potential anaphylactic saponin at different concentrations (mM). For a specific saponin, the cell viability (average) of the control groups was set to 1.

|  | 0 | 0.012 | 0.048 | 0.12 | 0.24 | 0.48 |
| --- | --- | --- | --- | --- | --- | --- |
| noto-ST-4 | 1.0 | 0.90 | 0.32 | 0.078 | 0.060 | 0.057 |
| Rg3 | 1.0 | 0.87 | 0.34 | 0.048 | 0.035 | 0.024 |
| Rh4 | 1.0 | **-** | 0.75 | 0.062 | 0.075 | 0.060 |
| Rk3 | 1.0 | **-** | 0.80 | 0.27 | 0.075 | 0.11 |
| gyp-LXXV | 1.0 | **-** | 0.87 | 0.60 | 0.080 | 0.12 |
| noto-T5 | 1.0 | **-** | 0.73 | 0.58 | 0.074 | 0.084 |


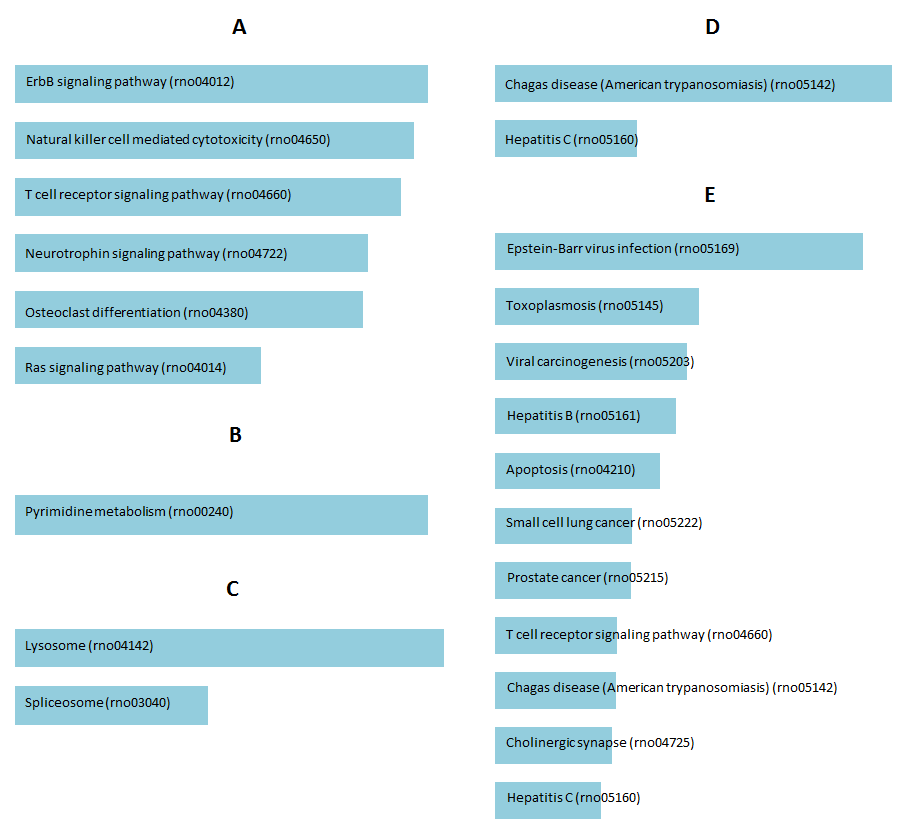


**Figure S2** KEGG enrichment pathway analysis results of the (A) noto-ST-4 groups, (B) Rg3 groups, (C) Rh4 groups, (D) Rk3 groups and (E) noto-T5 groups based on the significantly upregulated and downregulated proteins. Longer bars mean lower p-values.
